# Problems of Pathogenesis and Pathogenetic Therapy of COVID-19 from the Perspective of the General Theory of Pathological Systems (General Pathological Processes)

**DOI:** 10.3390/ijms22147582

**Published:** 2021-07-15

**Authors:** Evgenii Gusev, Alexey Sarapultsev, Desheng Hu, Valeriy Chereshnev

**Affiliations:** 1 Institute of Immunology and Physiology, Ural Branch of the Russian Academy of Science, 620049 Ekaterinburg, Russia; gusev36@mail.ru (E.G.); v.chereshnev@iip.uran.ru (V.C.); 2 School of Medical Biology, South Ural State University, 454080 Chelyabinsk, Russia; 3 Department of Integrated Traditional Chinese and Western Medicine, Union Hospital, Tongji Medical College, Huazhong University of Science and Technology, Wuhan 200092, China; desheng.hu@hust.edu.cn

**Keywords:** ARDS, cytokine storm, general pathological process, low-grade inflammation, microcirculation, MODS, receptor scavengers, SARS-CoV-2, systemic inflammation

## Abstract

The COVID-19 pandemic examines not only the state of actual health care but also the state of fundamental medicine in various countries. Pro-inflammatory processes extend far beyond the classical concepts of inflammation. They manifest themselves in a variety of ways, beginning with extreme physiology, then allostasis at low-grade inflammation, and finally the shockogenic phenomenon of “inflammatory systemic microcirculation”. The pathogenetic core of critical situations, including COVID-19, is this phenomenon. Microcirculatory abnormalities, on the other hand, lie at the heart of a specific type of general pathological process known as systemic inflammation (SI). Systemic inflammatory response, cytokine release, cytokine storm, and thrombo-inflammatory syndrome are all terms that refer to different aspects of SI. As a result, the metabolic syndrome model does not adequately reflect the pathophysiology of persistent low-grade systemic inflammation (ChSLGI). Diseases associated with ChSLGI, on the other hand, are risk factors for a severe COVID-19 course. The review examines the role of hypoxia, metabolic dysfunction, scavenger receptors, and pattern-recognition receptors, as well as the processes of the hemophagocytic syndrome, in the systemic alteration and development of SI in COVID-19.

## 1. Introduction

At the end of 2019, a new coronavirus (CoV), a severe acute respiratory syndrome, coronavirus 2 (SARS-CoV-2), which causes coronavirus disease 2019 (COVID-19), emerged [1]. COVID-19 is a global public health hazard that has resulted in a pandemic in many nations [2]. The current emergence of COVID-19 is the third outbreak of CoV in humans in the past 2 decades, following severe acute respiratory syndrome (SARS) and the Middle East respiratory syndrome (MERS) in 2002 and 2012, respectively [3]. Humanity will probably continue to face new variants of CoV and other viral infections that are comparable in socio-epidemic significance to COVID-19. This determines the need to take into account the typical patterns of this kind of infection using the example of COVID-19. At the same time, already today, from the standpoint of general pathology, three “large” general pathological processes can be distinguished that underlie the pathogenesis of COVID-19. First, there is chronic systemic low-grade inflammation (ChSLGI), which is a typical pathogenetic platform for many chronic diseases associated with old age and metabolic dysfunctions. Simultaneously, several disorders associated with ChSLGI are risk factors for severe complications of COVID-19. Second, these disorders are both local and systemic phenomena of classical inflammation associated with a predominantly local response (in the foci of inflammation and lymphoid organs) to an infection of innate and adaptive immunity. Third, there is life-threatening systemic inflammation (SI), which clinically manifests in the forms of systemic microcirculatory disorders, disseminated intravascular coagulation (DIC), and various complexes of resuscitation syndromes, including multiple organ dysfunction syndrome (MODS), DIC syndrome, and acute respiratory distress syndrome (ARDS) [4]. The mechanisms of cellular and tissue pro-inflammatory stress constitute the common core of their pathogenesis [5]. The fundamental regularities of these three general pathogenic processes, connected in COVID-19, are nonspecific to the etiological cause. 

Overall, the COVID-19 challenge highlights the importance of recognizing broad patterns in the pathophysiology of critical illnesses and developing novel methodological approaches for their pathogenetic therapy. This review will look at the causes and consequences of systemic damage and immune responses, which are the leading causes of death in COVID-19.

## 2. Systemic Reactions in COVID-19

Alveolocytes (particularly type 2), alveolar macrophages, and endothelial cells of the microvessels of the lungs are the first targets of the SARS-CoV-2 virus [6]. Angiotensin-converting enzyme 2 (ACE2), a key regulator of the renin-angiotensin system, serves as the functional receptor of SARS-CoV-2 [7]. Unlike its homolog ACE, ACE2 promotes the neutralization, rather than the production, of the vasopressor and pro-inflammatory cytokine Ang II, converting it into Ang 1-7, a vasodilator and anti-inflammatory cytokine [8,9].

ARDS is the most common critical complication of COVID-19 pneumonia and one of the leading causes of death [10,11,12,13,14]. While comprehensive data will be received in the future, nowadays, approximately 20% of hospital-acquired pneumonia with SARS results in ARDS [15]. In turn, ARDS leads to the development of critical hypoxia and SI—and, as the clinical reflection of SI—to various complexes of resuscitation syndromes [14,16,17].

Old age, obesity (related to insulin resistance), metabolic cider, type 2 diabetes, hypertension, and other cardio-vascular illnesses are the key risk factors for critical illness in COVID-19 [18,19,20,21]. ChSLGI, which is widespread in the vascular system, is their shared systemic pathogenetic nucleus [22]. In general, COVID-19 severe symptoms are more common in older patients with underlying cardiac, pulmonary, and metabolic disorders, notably those complicated by ARDS [23]. COVID-19, in turn, is a complicating factor for other critical diseases, such as burn injury and surgical complications [24,25].

### 2.1. Signs of Acute Phase Response and Hypercytokinemia in COVID-19

According to the study on infected human peripheral blood mononuclear cells, the non-structural proteins nsp9 and nsp10 of SARS-CoV-2 target NKRF (a repressor of the pro-inflammatory transcription factor NF-KappaB), boosting the production of IL-6 and IL-8 [26]. Furthermore, when SARS-CoV-2 infects target cells, aberrant activation of the NLRP3 inflammasomes, promoting the production of IL-1β and IL-18, as well as pyroptosis (inflammasome-dependent programmed cell necrosis) is observed [27]. With persistent SARS-CoV2 infection in non-critical hospital patients, a moderate but long-term increase in IL-6, TNF-α, IFN-γ, IL-2, IL-4, and IL-10 cytokines during the development of cellular and humoral immune responses can be seen [28].

The viral load of SARS-CoV-2 has been linked to blood levels of IL-6 in COVID-19 patients, suggesting that this cytokine may have a role in the development of ARDS [29]. With that, the comparison of blood levels of IL-1, IL-1RA, IL-6, IL-8, IL-18, and TNF-a in ARDS COVID-19 and bacterial sepsis revealed no significant changes [30]. Increases in the chemokines CXCL10, CCL7, and IL-1 in the blood were linked to illness severity, and CXCL10 levels were the only ones that positively and substantially correlated with viral load [31]. It is important to remember that large-scale alterations in the cytokine network at the whole-organism level are not possible without the involvement of additional inflammatory mediators, such as diverse classes of eicosanoids (“eicosanoid storms”), as seen in COVID-19 [32]. On the other hand, the changes in cytokine and acute-phase protein concentrations in the blood are critical for determining the pathogenetic and prognostic relevance of the systemic inflammatory response (SIR). In this regard, assessing the variations in these variables in patients receiving intensive care versus those with a milder course of COVID-19 is of particular interest. Currently, a large number of studies [33,34,35,36,37,38,39,40,41,42,43,44,45,46,47,48,49,50,51,52,53,54,55,56] are devoted to these distinctions. The results of these studies are summarized in Table 1, which allows us to draw several conclusions regarding the increase in SIR indicators with an increase in the severity of COVID-19.

SIR tends to rise as the patient’s condition worsens due to both the acute phase response (ferritin, CRP) and hypercytokinemia, which is associated with an increase in the levels of both pro-inflammatory (chemokines, especially CXCL10) and anti-inflammatory (IL-10 and IL-1ra) cytokines, and cytokine factors of lymphopoiesis (IL-7, and in some cases, IL-2).

The increased levels of cytokines of the second type of immune response (Th2 cells, M2a macrophages, eosinophils, basophils, mast cells, and IgE anti-bodies) are not typical of COVID-19 complications, including IL-4, IL-5, IL-13, and Eotaxin.

The differences in pleiotropic IL-6, rather than TNF- and IL-1, are more obvious among the proinflammatory cytokines, and the increase in IFN- is delayed.

The change in cytokine levels in the blood is non-linear in general, with an irregular distribution, and the predictive usefulness of even the most studied cytokines (IL-6 and IL-10) is not confirmed.

The contradictory results of these studies can be explained by the following:

(a) It is crucial to separate cases of stage 2–3 ARDS, DIC syndrome, rapidly progressing MODS, and refractory shock among patients in critical care units, which are clinical equivalents of SI as a special form of the general pathological process [5,57];
(b) It is important to consider SI dynamics, such as the presence of hyperergic and hypoergic (depressive) phases [5,57];
(c) It is necessary to account for the instability of individual SIR indicator concentrations in the blood (especially cytokines) and use for this integral SIR criteria with the preliminary establishment of specific levels of pathobiological and diagnostic significance for each SIR indicator, followed by their integration into a single scale [4,5,57].

### 2.2. The Main Factors of Systemic Alteration in COVID-19

Intravascular activation (for various reasons) of the complement and hemostasis systems, intravascular leukocytes, vascular endothelium including vascular macrophages, and particularly the Kupffer cells of the liver sinus capillaries are the main causes of SI as a critical complication. The main causes of systemic pro-inflammatory stress, which is crucial for the patient’s survival, are:

(a) Systemic hypoxia (an obvious phenomenon in ARDS);
(b) Entry of tissue factor (TF) into the bloodstream or increased expression of its membrane form (CD142) on the endothelium’s surface, entry of other hemostasis and complement activators into the blood; accumulation of microbial-pathogen-associated molecular patterns (PAMP) and endogenous-damage-associated molecular patterns (DAMP) in blood, acting on cells through pattern-recognition receptors (PRR) [5];
(c) Systemic activation relationship of leukocytes and macrophages despite the loss of their antiviral efficacy [58];
(d) The entry into the bloodstream of a significant number of inflammatory mediators with the development of the phenomenon of excitotoxicity.

The term “excitotoxicity” refers to the toxic effects of activation factors (including cytokines) on central nervous system neurons [59,60,61]. In patients with hypercytokinemia, however, excitotoxicity manifestations may have a broader pathobiological significance. This phenomenon can serve as a positive feedback loop for a SI that has already begun to develop. As a result, the local occurrence of “cytokine storm” in the lungs could be a significant cause of the development of ARDS in COVID-19, important for the launch of SI [62].

#### 2.2.1. Hypoxia

One of the main reasons for the development of SI and mortality in COVID-19 is hypoxemia linked with respiratory failure and systemic tissue hypoxia associated with several causes of impaired oxygen transport [63,64,65,66].

Similarly, hypoxemia/hypoxia is thought to be an independent risk factor for hospital mortality in the most critical forms of ARDS [63,64,65,66], as it is linked to the development of multiple organ failure and coagulopathy [63,64,65,66]. COVID-19 patients frequently develop hypoxemia without signs of breathlessness though their chest X-rays show diffuse pneumonia, and dangerous levels of blood oxygen are detected [63]. Meanwhile, low saturation values cause cascades of molecular changes in many tissues, which can trigger a chain of events that result in organ damage that can be fatal [63].

Hypoxia as a damaging factor simultaneously stimulates numerous cellular stress signaling pathways, primarily those involving Hypoxia-Inducible Factors (HIF) [67], NF-KappaB, and mitogen-activated protein kinases (MAPK) [68,69,70]. In turn, viral infection and obesity are independent factors that raise HIF-1 expression, exacerbating the production of pro-inflammatory cytokines and raising the risk of cytokine storm [65]. As a result, increasing hypoxia amplifies the COVID-19 disease process, contributing to the establishment of vicious pathogenetic circles alongside hyperinflammation and coagulopathy. [66]. 

Despite some unique characteristics in COVID-19 tissue, hypoxia, linked with hypoxemia and reduced oxygen delivery, is a common occurrence governed by universal laws. The pathogenic significance of systemic hypoxia in uncontrolled hemorrhagic (hypovolemic) shock is revealed by cytokinemia levels that are comparable to those in septic shock [4]. Hypoxia can directly trigger DIC and critical microcirculatory disorders. HIF, in particular, promotes the expression of tissue factor (TF) and plasminogen activator inhibitor-1 (PAI-1) while suppressing the expression of tissue factor pathway inhibitor (TPFI), thrombomodulin, and protein S, which could lead to the development of DIC [65]. Even in the absence of hypotension or blood loss, hypoxia causes numerous populations of macrophages and other cells to activate, synthesize, and release proinflammatory cytokines, resulting in hypercytokinemia and substantially elevated levels of circulating TNF- and IL-6 [71]. HIF-1 also promotes the expression of NLRP3 in a variety of inflammasome cells linked to IL-1 production and pyroptosis [72]. Nuclear transcription factors can be transferred from activated cells to distant organs via extracellular microvesicles circulating in the blood under hypoxia [73]. Hypoxia, in general, causes pro-inflammatory cellular stress in cells, including the immune system, with the goal of cell survival, tissue homeostasis preservation, and the restoration of damaged structures [5,74]. However, the involvement of hypoxia and HIF-1 in cytokine expression regulation is controversial and depends on a variety of factors. Hypoxia and HIF-1 thus can activate or inhibit the inflammatory response mediated by cytokines [75]. Hypoxia, as with any other cause of pro-inflammatory cell activation, is characterized by tissue stress, losing its adaptive value under conditions of excessive duration, the severity, and prevalence of the harmful factor’s action, and then “the treatment becomes worse than the disease”. This also holds for the effects of transmitted tissue stress. Surviving cells can have the phenotype of accelerated aging after experiencing acute hypoxia, such as hemorrhagic shock [76,77]. It is important to remember that several mechanisms for cellular stress development can be combined. Not just hypoxia, but also other pro-inflammatory stimuli, such as the influence of Gram-negative bacteria’s lipopolysaccharide (LPS) on cells, can activate HIF-1. Hypoxia, in turn, can amplify the deleterious effects of other damaging factors on cells, including through similar signaling pathways [78,79,80,81]. As a result, systemic hypoxia can be one of the reasons for systemic change, SI, and critical conditions in COVID-19 patients with ARDS. Furthermore, it should be noted that type 2 diabetes mellitus, which is a common comorbid pathology in COVID-19, increases HIF-1 expression in cells [82].

#### 2.2.2. Cell Activation by Pattern-Recognition Receptors

The accumulation of PRR ligands in the systemic circulation, notably PAMP and DAMP, is another clear rationale for the development of SI in COVID-19. When PRR is activated, cells undergo a quick and significant pro-inflammatory response, primarily innate immunity [83,84]. These mechanisms, in particular, can rapidly trigger cellular proinflammatory change associated with autophagy and hyperactivation of NF-KappaB transcription factors [85]. These modifications can cause a pro-inflammatory phenotype in cells as well as different types of programmed cell necrosis (necrobiosis, pyroptosis, and NETosis—NET, Neutrophil Extracellular Trap) [86]. In contrast to apoptosis, DAMPs penetrate the intercellular milieu during necrosis, which can lead to SI’s uncontrolled self-development in certain situations. Other factors that function in concert with PRR, such as cytokines, hypoxia, and activated platelets, can trigger NETosis of phagocytes, the most traumatic for the surrounding tissues [87]. Due to the combination with a secondary infection or disturbance of the microbiota and barrier integrity of the gut during SARS-CoV-2 invasion, SARS-CoV-2 antigens can operate directly as a PAMP, but so can structures of other microorganisms, including LPS [88,89]. The source of DAMP, on the other hand, is tissue breakdown products, various secretion products, and stress-cell membrane expression. [5].

SARS-CoV-2 is hypothesized to directly bind the primary PRR for LPS, Toll-like receptors 4 (TLR4), and activate TLR4 signaling to enhance ACE2 expression on the alveocyte surface, enabling SARS-CoV-2 penetration and contributing to the development of ARDS [90]. At the same time, it is suggested that the activation of TLR4 and consequent overactivation of the innate immune response may be linked to myocarditis triggered by SARS-CoV-2, as well as other organ damages in COVID-19. Thus, the molecular docking studies have demonstrated significant binding of the native SARS-CoV-2 protein-S to TLR1 and TLR6, although with lower binding energy than with TLR4 [91].

#### 2.2.3. Metabolic Causes of Tissue Damage, the Possible Role of Chronic Low-Grade Inflammation in the Pathogenesis of COVID-19

On the cell surface, PRRs typically form functional complexes with PRRs from the same class (for example, TLR1, TLR2, and TLR6) as well as receptors of a different nature, primarily tetraspanins, integrins, and several types of scavenger receptors (SR) [92]. In this scenario, SR is at the crossroads of immunity, inflammation, and metabolism, with a wide range of ligand specificity and functionality. PAMP and DAMP, changed cells and platelets from the body (damaged, apoptotic, senescent, malignant, pathologically activated), as well as several transformed metabolites, such as oxidized low-density lipoproteins (ox-LDL), denatured, and glycated proteins [92,93], can all operate as SR ligands. The fact that the rise in SR expression on various cells is directly proportional to the number of their ligands is one of the common aspects of SR. They participate in the cleansing of tissues by macrophages from metabolic and cellular “debris”. Furthermore, several SRs can modulate the intensity of proinflammatory cellular stress in various ways. These characteristics of SR predetermine their involvement in the establishment of allostasis, or long-term altered homeostasis [94]. Systemic metabolic illnesses such as aging, morbid obesity, metabolic syndrome, and type 2 diabetes are all known to cause allostasis. These pathologies are the risk factors for severe complications of COVID-19.

The following observations rule out the idea of SR involvement in the pathophysiology of COVID-19 critical complications. Some SR, such as CD209 [95] and SR-B1 [96], have the ability to bind SARS-CoV-2 and participate in the virus’s entry into the cell. In sepsis, the pathogenetic relevance of SR has been demonstrated. Thus, it has been shown that blocking SR-E1 (LOX-1) on endotheliocytes or lowering its primary ligand in the blood, oxLDL, increases the survival rate of mice with experimental sepsis [97]. The major receptor for advanced glycation end products is SR-J1 (RAGE) (AGE). Hyperglycemia enhances RAGE expression in a variety of cells (including endothelial cells, vascular macrophages, myocytes, and cardiomyocytes) [92]. RAGE can bind a variety of PAMP and DAMP (including S-100, HMGB1), oxLDL, and amyloid proteins, activating cells in the same way that standard PRR does [98]. The presence of SR-J1 (RAGE) on endothelial cells and blood leukocytes is an unfavorable factor in the development of septic shock, other critical situations, as well as diabetic vascular problems [99,100]. At least three soluble forms of SR in the blood, notably sCD14 (presepsin) [101], sSR-I1 (sCD163) [102], and sSR-E3 (sCD206, mannose receptor 1) [103], are useful markers for the verification or prognosis of critical conditions associated with infection and other causes. Simultaneously, the usefulness of sCD14 (TLR4 coreceptor) for the prognosis of severe sequelae in COVID-19 has been demonstrated [104,105]. Plasma sCD163 levels measured early in the course of the disease may be useful in predicting the severity of COVID-19 pneumonia [106]. The level of sCD163 in the blood rises dramatically when the patient progresses to the development of ARDS COVID-19. Haptoglobin–hemoglobin complexes and several PAMPs bind to CD163. Depending on the type of their activation, they are only expressed on macrophages and monocytes.

Glucocorticoids, IL-6, IL-10, and heme/Hb are the most potent stimulators of CD163 expression, whereas IL-4, GM-CSF, IFN-, TNF-, CXCL4, and LPS inhibit CD163 expression [107]. Furthermore, in the comorbidity of acute and severe chronic conditions, including type 2 diabetes, sCD163 is a predictor of mortality risk [108]. The content of sSR-E3 (sCD206) can be used to predict complications of community-acquired pneumonia [109]; however, the significance of this marker in COVID-19 remains unclear. The research of the SR-B2 (CD36) receptor in COVID-19 is promising [5,93]. CD36 can form receptor complexes on the cell surface (endotheliocytes, macrophages, platelets, erythroid cells) with 1, 2, 5 integrins, TLR (TLR2, TLR4, TLR6), Fc receptors for antibodies—IgG (FcR), tetraspanins CD9 and CD81 after contact with ligands (PAMP, oxLDL, free fatty acids (FFA), and others after contact. It is involved in the creation of the CD36-TLR4-TLR6 complex as well as an integral signaling pathway (including MAPK and NF-KB) by which atherogenic lipoproteins and b-amyloids induce an aseptic pro-inflammatory response in macrophages, including microglial cells [110]. Moreover, atherosclerosis, neurodegeneration, coagulopathy, sludge syndrome, and the development of microcirculatory diseases are also all linked to the CD36 receptor [111,112,113,114] and seen in COVID-19.

Insulin resistance is defined as a reduction in tissue insulin responsiveness due to reduced insulin receptor expression and blockage of insulin signaling pathways in a variety of cells (mostly in the liver, muscles, and adipose tissues). The development of insulin resistance in obesity is associated with the regulatory and metabolic effects of FFA, an increase in the adipose tissue of macrophages, fibroblasts, and the pro-inflammatory status of the fat depot in general [115,116,117,118]. Insulin resistance stabilizes metabolic imbalances and ChSLGI allostasis, which are common in metabolic syndrome and type 2 diabetes. Overproduction of pro-inflammatory cytokines (including TNF- and IL-6) in adipose tissue, as well as an imbalance between adipokines that increase or reduce tissue sensitivity to insulin (adiponectin and resistin) [119], are typical of insulin resistance disturbances. An overabundance of saturated FFA, cholesterol, lysophosphatidylcholine, and ceramides in the blood, on the other hand, can act as a lipotoxicity factor [120,121,122,123,124,125]. As one of the FFA transporters, SR-B2 (CD36) may also play a role in the buildup of intracellular lipids and the development of lipotoxicity [126,127].

Lipotoxicity linked with a rise in FFA, according to several studies, is the primary cause of type 2 diabetes [128,129,130]. Hypoxia, on the other hand, can increase lipotoxicity and pro-inflammatory macrophage activation by saturated FFAs [80]. In acute systemic manifestations of inflammation, such as sepsis, high levels of FFA in plasma can be one of the causes of heart damage [80]. The action of oxLDL on macrophages in COVID-19 increases uncontrolled cytokine production and alveolar injury [131]. Antiviral activity was found in native high-density lipoprotein (HDL) with antioxidant and antiatherosclerotic activity, which inhibited SARS-Co-V2 replication, but glycated or oxHDL lost its antiviral action [132]. In COVID-19, metabolic status changes, namely, the increased levels of circulating glucose and FFA, high levels of oxidative stress, and SIR (including IL-6, CRP), that are characteristic of pathologies associated with insulin resistance and correlates with the severity of the disease, may occur [133]. Insulin levels are often increased in sepsis patients, while insulin sensitivity is decreased [134]. The increased blood levels of FFA and native lipoproteins, on the other hand, are not a common indicator of critical conditions, including sepsis. As a result of a lack of the enzyme Lecithin-cholesterol acyltransferase and other factors, which is associated with the activation of hyperergic processes and disturbances in the formation and functioning of lipid transport forms [135], the opposite tendency is frequently observed in sepsis [135,136,137,138].

In sepsis, however, determining the increase in blood FABP4/A-FABP (fatty acid-binding protein 4) [139] appears to be more important. A-FABP is an intracellular carrier of FFA but can be secreted into the blood by activated adipocytes, especially in obesity. In this case, extracellular A-FABP can be associated with the expression of TLR, activation of macrophages, synthesis and release of TNF-α and IL-6, and the synthesis of eicosanoids. Blood levels of A-FABP > 40 ng/mL are associated with poor outcomes in critical conditions [140]. Furthermore, a high A-FABP concentration has been linked to obesity, type 2 diabetes, and cardiovascular disease [140]. The expression of A-FABP is stimulated by FFA, oxLDL, LPS, and AGE [141].

A drop in the blood polyunsaturated/saturated FFA ratio is an unfavorable trend in sepsis [142]. OxLDL levels rise in severe sepsis and septic shock, often in the face of a drop in native LDL [143,144]. Saturated FFAs have been shown to activate TLR4 in adipocytes and other cells [145]. TLR4 activation, in turn, promotes insulin resistance and the deposition of triacylglycerol in the liver in mice who consume too much fructose [146]. FFAs bind to TLR4 indirectly through the involvement of FFA-binding plasma proteins. Fetuin-A (FetA), which regulates insulin sensitivity in the liver via TLR4 signaling, can serve this role in mice (and possibly other species and humans) [147]. The liver secretes FetA glycoprotein, which is the major transporter of FFA in the bloodstream and a pleiotropic regulator of proinflammatory stress in several organs; it can contribute to lipotoxicity in pancreatic cells via the TLR4-JNK-NF-KB signaling pathway [148].

As a result, metabolic dysfunction is linked to other sources of systemic pro-inflammatory stress, including bacterial sepsis and COVID-19, not only in chronic disorders but also in acute critical conditions. However, the pathogenetic significance of metabolic dysfunctions in CoV infections is currently understudied, making it difficult to determine the impact of comorbid diseases as risk factors for COVID-19 severe sequelae.

#### 2.2.4. Tissue Damage by Activated T Cells and Macrophages; Assessment Issues in COVID-19 Cytokine Release Syndrome and Cytokine Storm Syndrome

The most obvious manifestations of COVID-19 hypercytokinemia are presently classified as cytokine release syndrome (CRS) and cytokine storm syndrome (CSS). Both syndromes are identified in several autoimmune and tumor diseases, congenital autoinflammatory syndromes [149], and are related to hypercytokinemia in response to infection and certain types of immunotropic therapy. CSS, on the other hand, is associated with faster dynamics and, in general, higher blood levels of pro-inflammatory cytokines. Pathological activation of T cells (particularly CD8 + T-killers and Th1), as well as malfunctioning of normal killer cells (NK), secondary activation, and involvement in cytokinogenesis (IL-1, IL-6, TNF-, IL-8, IL-10) monocytes/macrophages, are thought to be the main causes of CRS/CSS [150]. Thus, the treatment with CAR-T-cells (chimeric antigen receptor T-cells) or anti-ti-lymphocytic antibodies in several lymphoproliferative and tumor diseases is a typical pharmacological cause of CRS/CSS [151,152]. Due to the loss of control from negative feedback regulatory mechanisms, there may be a targeted blockage of T cells’ antiviral function in viral infections accompanied by an increase in their phlogogenic activity. However, the causes of SIR in COVID-19 are polyvalent, as previously stated, and identifying the fundamental and defining T-cell mechanism of systemic alteration is challenging. Furthermore, there is currently no uniform diagnostic standard for COVID-19 in relation to CRS/CSS. The occurrence of atrophy of the secondary lymphoid organs is a distinguishing hallmark of COVID-19’s “cytokine storm”. In contrast, lymphadenopathy and splenomegaly are rarely seen in other CSS-related disorders [149].

CSS in COVID-19, as with other disorders, is thought to be linked to specific clinical criteria, specifically hemophagocytic lymphohistiocytosis (HLH) [153]. Macrophage activation syndrome (MAS) is a term used by certain authors to describe the secondary HLH [154,155]. Patients with MAS criteria or concomitant hepatobiliary dysfunction + DIC + MODS (MAS-like syndrome) account for 3–4 percent of sepsis patients [156].

Acute, persistent fever, lymphadenopathy, hepatosplenomegaly, MODS, and CSS are the most prevalent clinical symptoms of HLH [157]. CD163 + macrophages are generated during hemophagocytosis and demonstrate compensatory anti-inflammatory action. Very high blood ferritin levels are a particular sign of MAS: a level of 10,000 ng/mL has 96 percent specificity and 90 percent sensitivity for MAS [157]. At least 5 out of 8 symptoms (fever, splenomegaly, bicytopenia, hypertriglyceridemia and/or hypofibrinogenemia and hemophagocytosis, low/absent NK activity, hyperferritinemia, and high levels of sIL-2R/sCD25) are required to make a diagnosis according to the HLH-2004 criteria, of which only 6 are commonly available in clinical practice [158]. Meanwhile, an independent study of 19 severe COVID-19 cases with symptoms of hyperinflammation (neutrophil/lymphocyte ratio > 3.5) found that none of the patients met more than three HLH-2004 criteria [159]. For HLH/CSS verification and COVID-19 [160], the modified HLH-2009 criteria can be used. However, these criteria have limited sensitivity for assessing COVID-19 critical situations, and the levels of cytokines in these settings frequently make it difficult to distinguish them from adaptive cytokinemia in uncomplicated classical inflammation [161]. Furthermore, despite some tentative favorable outcomes [162], subsequent randomized studies of COVID-19 treatment with anti-cytokine therapy, such as anakinra (IL-1 receptor antagonist) and tocilizumab (monoclonal IL-6 receptor antibody) [163,164,165], have shown negative results. Tocilizumab, in combination with glucocorticoids, produces slightly better results, but only at particular stages of the development of COVID-19 problems (in patients who do not require mechanical ventilation) [161]. Antibodies against TNF-α seem to be of modest effect for patients with SARS-CoV-2/ARDS infections, according to preliminary reports [166]. These findings are consistent with the mixed effects of anti-cytokine therapy in different sepsis types [167,168].

As a way out of this situation, the “milder” CSS prediction criteria for COVID-19 were established, which included four groups of laboratory results: the existence of an acute phase response (ferritin > 250 ng/mL and CRP > 4.6 mg/dL), as well as the presence of at least one feature from three clusters: (1) albumin <2.8 g/dL, lymphocytes (%) < 10.2, neutrophil abs > 11.4 K/mm3); (2) ALT > 60 U/L, AST > 87 U/L, D-dimers > 4.930 ng/mL, LDH > 416 U/L, troponin I > 1.09 ng/mL; (3) anion gap < 6.8 mmol/L, chloride > 106 mmol/L, potassium > 4.9 mmol/L, BUN: creatinine ratio > 29 ratio [169]. With that, none of the CSS criteria presented is directly related to cytokines. Furthermore, it is unclear how such a cutoff threshold for acute phase response markers in COVID-19 might characterize the criticality of SIR. It is also uncertain how an acute phase response, in combination with indications of lymphopenia/neutropenia, tissue damage, DIC, and alterations in electrolyte balance, might characterize the fundamental pathogenic connection of HLH/MAS, namely, T cell dysfunction, NK activation, and macrophage pathological activation. Meanwhile, it is worth noting that the clear morphological markers of HLH in COVID-19 patients, such as hemophagocytosis and lymphoproliferative processes in the bone marrow and secondary lymphoid organs, are rarely discovered during postmortem examination.

The causes of COVID-19 hypercytokinemia are numerous. Excitotoxicity is caused by a number of variables, including cytokines, although they are not the only ones. Excitotoxicity is not the only factor that contributes to the onset of SI. During the expansion of SARS-CoV-2 in the lungs, cytokines can be produced not only systemically but also locally by alveolar macrophages and other cells at the site of inflammation [170]. The mechanisms of secondary HLH (MAS) or HLH-like conditions may be involved in the complex of causal variables in the development of SI in COVID-19. However, we consider that there is not enough adequate evidence to exclusively link the “cytokine storm” to these pathways, much less to accept CSS as the primary cause of critical circumstances in all COVID-19 variations. Rather, we are seeing a shift in the way CRS and CSS are interpreted, with these syndromes evolving from clinical definitions to conceptual quasi-syndromes that serve as surrogates for general pathological processes. Thus, the SIR syndrome (SIRS) served as a surrogate for a long time before the introduction of the most recent version of sepsis (Sepsis-3) in 2016 [171,172].

The lack of widely agreed empirical guidelines for identifying the phenomena of hypercytokinemia is the most obvious problem with CRS/CSS verification. This is due to the increasing chaos, abnormality, and nonlinearity of changes in the levels of even the most diagnostically important cytokines, as well as their reliance on genetic, ontogenetic, and other patient characteristics, treatment, comorbidity, and process dynamics, and research methods used during the development of critical conditions. According to our findings, the absolute critical values of IL-6 are less than 1000 pg/mL [4,57]. This statement is supported by some other studies as well [173,174]. However, such high amounts of IL-6 in sepsis are uncommon. The standard cutoff thresholds for IL-6 concentration in blood plasma for confirming a severe course of COVID-19 and serious sepsis vary from 20 to 450 pg/mL, with a norm of IL-6 of 5–7 pg/mL [42,52,175,176]. Such hypercytokinemia criteria, on the other hand, have only probabilistic significance for the verification of SI and predicting the prognosis of critical complications. At the same time, considerably lower and sometimes normal levels of IL-6 have been found in some patients during the crucial phase of COVID-19. To tackle this difficulty, we believe that integral SIR criteria, which include at least 5–6 markers of acute phase response and cytokinemia (perhaps other SIR variables) [57], should be used. Simultaneously, in addition to the existence of diagnostically substantial levels of SIR, other aspects of this general pathological process must be determined to describe SI [4].

**Figure 1:**
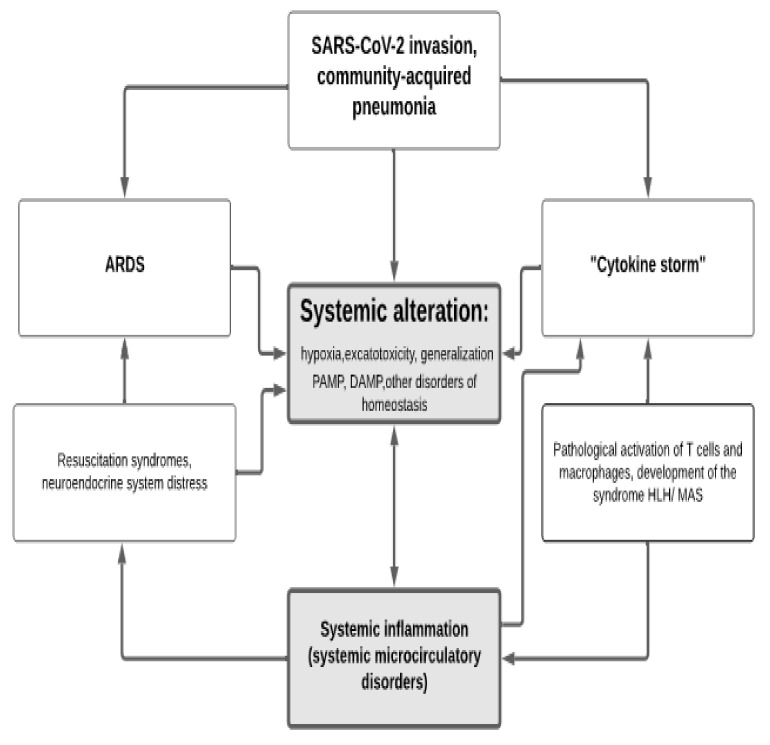
Transformation of classic inflammation in COVID-19 into systemic inflammation with the formation of a vicious pathogenetic circle. Three key mechanisms may be distinguished in the formation of a vicious pathogenetic circle and the development of a critical state in COVID-19: (1) the severity of ARDS (Acute Respiratory Distress Syndrome), which is linked to acute lung damage and systemic microcirculatory abnormalities, regardless of the primary focus of damage. Additionally, with the development of other resuscitation syndromes such as DIC and MODS, the phenomenon of systemic change promotes the onset of organ dysfunctions and coagulopathy. (2) During the development of microcirculatory diseases, DAMPs (damage-associated molecular pattern) and TF (Tissue Factor) and other hazardous products of tissue degradation are widespread. (3) The emergence of the “cytokine storm” phenomenon, which is linked to hemophagocytic lymphohistiocytosis (macrophage activation syndrome, MAS) and overproduction of cytokines in the focus of COVID-19 canonical inflammation, sharply increases with the generalization of pro-inflammatory tissue stress linked to SI.

In general, various pathways of systemic alteration lead to the development of SI and microcirculatory disorders, which in turn drive the phenomenon of secondary systemic damage. Ultimately, this leads to additional accumulation of DAMP and PAMP in the blood. Furthermore, systemic activation of endothelial cells, vascular macrophages, blood leukocytes, and other cells can dramatically increase the phenomena of “cytokine storm” and the excitotoxicity that goes along with it. These changes, taken collectively, result in secondary systemic harm and the formation of vicious pathogenetic loops (Figure 1). The supportive environment for these processes is formed by metabolic abnormalities and insulin resistance, which coexist with central obesity, metabolic syndrome, and type 2 diabetes. All of these phenomena have been reported in critical conditions of diverse etiologies, as well as life-threatening viral infections such as COVID-19.

### 2.3. Endothelial Dysfunction and Microcirculatory Disorders in COVID-19

Endothelial damage and concomitant microcirculatory abnormalities, as well as major vessel thrombosis, are a common pathogenetic basis of pathologies of internal organs in a variety of critical conditions, including severe forms of COVIID-19. Simultaneously, the endothelium’s vulnerability in COVIID-19 may increase in the presence of comorbid disorders such as type 2 diabetes [177]. The central symptom of ARDS is pulmonary microvascular barrier dysfunction and uncontrolled exudation, and systemic microcirculatory abnormalities, which are strongly linked to DIC and other manifestations of SI, act as a key mechanism for the development of MODS and shock conditions [178,179]. D-dimers appear to be one of the most useful DIC markers for predicting COVID-19 critical complications [180]. Simultaneously, one study found that unequivocal symptoms of DIC syndrome were present in 71.4 percent of those who died and only 0.6 percent of those who survived severe pneumonia COVID-19 [181]. In this situation, microthrombus production is linked to a reciprocal activation of the endothelium. Thrombin, for example, is primarily involved in clot formation, but it also promotes inflammation by activating proteinase-activated receptors (PAR) on endothelial cells, particularly PAR1 [182]. Therefore, it is not surprising that there’s a link between the severity of COVID-19 and deaths, as well as the severity of markers of endothelial dysfunction and paracoagulation [183,184]. Despite the presence of the ACE2 receptor and its cofactor TMPRSS2 receptor on human platelets, the possibility of direct activation of SARS-CoV-2 platelets in DIC and thrombosis, as well as the use of aspirin or other non-steroidal anti-inflammatory drugs to relieve these complications in COVID-19, remains unclear [185].

Damage to the endothelial glycocalyx and subsequent activation of endothelial cells, the hemostatic system, and the development of systemic oxidative stress are all linked to microcirculatory disorders [186]. There is a paradoxical decrease in nitric oxide (NO) production due to inhibition of constitutive endothelial NO synthase (eNOS) in the microvascular network with relatively moderate oxidative stress and systemic pro-inflammatory condition [187,188]. While increasing the synthesis of vasopressors such endothelin-1 and Ang II, these alterations contribute to hypertension, which is a characteristic of ChSLGI. The inducible form of NO-synthase (iNOS) is triggered in response to systemic inflammation, which is accompanied by increased expression of NF-KappaB and other pro-inflammatory pathways [189,190]. This causes pathological vasodilation of arterioles and capillary sphincters, as well as a sudden drop in organ perfusion, hypoxia, and serious metabolic disturbances [191,192].

Numerous biochemical mechanisms, including iNOS expression and unregulated NO production, have been confirmed to start a cascade of inflammatory events in COVID-19 infection [193]. These alterations in COVID-19 may be linked to the STAT1/IRF1 signaling pathway, which is activated by the combined action of TNF- and IFN- in macrophages, resulting in the activation of iNOS and various forms of programmed cell death (pyroptosis, apoptosis, and necroptosis) [194]. HIF-1 can activate the expression of iNOS during systemic hypoxia (typical of COVID-19/ARDS), which can lead to microcirculatory abnormalities and cause systemic alterations of internal organs [195]. Endotoxemia coupled with poor intestinal barrier function can also result from iNOS activation and the formation of microcirculatory disturbances [196].

In the absence of a disease, the phenomenon of intravascular activation of phagocytes, primarily neutrophils, with the release of extracellular DNA in combination with various phlogogenic proteins capable of damaging the vascular endothelium is also functionally associated with microcirculatory disorders [197,198]. Even though practically all phagocytic leukocytes can form NETs (NETtosis), hyperactivated neutrophils play a crucial part in this process [5]. Therefore, there is no dispute about the presence of considerable NET formation in severe COVID-19 patients against the backdrop of systemic oxidative stress and other SI symptoms in the intravascular environment [199,200,201,202]. The formation of NET in COVID-19 was linked to the accumulation of paracoagulation factors, tissue damage, and pro-inflammatory neutrophil degranulation in the blood [203]. The formation of platelet–leukocyte aggregates or the activation of immune cells by soluble substances and active microvesicles released by platelets might cause this interaction [204]. The development of platelet–leukocyte aggregates and the adherence of these complexes to the injured endothelium could be the mechanisms of activation of intravascular phagocytes in this scenario [205,206]. The complement system and the kallikrein–kinin system, in turn, are intimately linked to endothelium activation and intravascular activation of the hemostasis system and leukocytes [5]. As a result, activation of the complement system, as well as the accumulation of anaphylaxin C5a in the blood, can be a marker of the severity of the illness in individuals with COVID-19 [207,208]. In the case of SARS-CoV-2 infection, cascade activation of plasma proinflammatory systems and vascular endothelium is also a common phenomenon [209].

Sublingual video microscopy revealed significant alterations in microvessels in COVID-19 patients. In patients with artificial lung ventilation, this method revealed a sharp decrease in vascular density (up to 90%) in the classes of vessels with a diameter of 4–10 m and thinning of the endothelial cell glycocalyx, which correlated with increasing levels of D-dimer and endotheliosis markers (VEGF-A, Angpt-2, TF, thrombomodulin), as well as some other indicators of SIR in the blood [55]. Critical conditions in COVID-19 are marked by a systemic pro-inflammatory transformation of the vascular endothelium, as evidenced by the accumulation of markers of endothelial cell activation and destruction of the endothelial glycocalyx in the blood, as well as an increase in syndecan-1 [186,210].

The Working Group on Atherosclerosis and Vascular Biology, together with the Council for Basic Cardiovascular Sciences of the European Society of Cardiology, has issued a Statement on the Importance of Endothelium in the Pathophysiology Underlying the Clinical Picture of COVID-19 [211]. The Statement includes evidence of endothelial cells’ involvement in SARS-CoV-2 infection, such as (1) the expression and function of the virus’s ACE2 receptor in the vasculature; (2) the prevalence of a syndrome similar to Kawasaki disease (vasculitis) among COVID-19 patients, and (3) evidence of SARS-CoV-2 infection of endothelial cells in COVID-19 fatal patients.

ACE2 and the cofactor for the receptor activity of ACE2 proteases are also expressed in endothelial cells of numerous organs, according to several studies [212,213,214]. Endothelial cells also express CD147 and CD209L, which may act as alternative receptors for SARS-CoV-2 entry [94,215]. Histopathological investigations of severe SARS-CoV-2 infections stress the importance of infection of microvessel endothelial cells, as well as the creation of micro- and macrovascular thrombosis in both venous and arterial blood flow [216]. The presence of viral particles in endothelial cells induces their death, which, together with the activation of systemic pro-inflammatory pathways of microvascular activation, could explain the systemic microcirculation dysfunction seen in COVID-19 patients [212,217,218]. In COVID-19 [219], one of the most common causes of thrombosis of big veins and great arteries is the presence of microcirculatory abnormalities and signs of paracoagulation. As the majority of the abnormalities listed above are typical, therapy for endothelial dysfunction in COVID-19 [220] can be based on those that have been validated in other systemic diseases.

### 2.4. Dysfunction of the Hypothalamic–Pituitary–Adrenal System (HPA)

HPA adaptation under stress may be insufficient to restore homeostasis: if the neuroendocrine system’s stress is excessive or insufficient in intensity or duration, we can talk about a state of maladjustment, a neuroendocrine system response to the type of distress that aggravates the patient’s condition [221]. After controlling for age, comorbidities, and laboratory tests, multivariate analysis revealed that doubling cortisol concentration in COVID-19 was associated with a substantial 42 percent increase in mortality risk [222], although there was no evidence of adrenal insufficiency in the acute phase of the disease. According to certain authors, the absence of severity of this problem renders the use of glucocorticoids in COVID-19 dubious [223]. At the same time, cortisol levels and the hospital anxiety and sadness scale (HADS) were considerably greater in COVID-19 patients who died than in survivors [224]. Other authors, on the other hand, found COVID-19 symptoms of adrenal insufficiency in the most severe COVID-19 patients despite low cortisol and ACTH levels [225]. Another study found that essential SARS-CoV-2 receptors, such as ACE2 and TMPRSS2, are co-localized in adrenal cells, and that cortisol levels are lower in COVID-19-positive critically ill patients than in COVID-19-negative critically ill patients [226]. Based on these findings, the authors advocate evaluating plasma cortisol levels for hormone therapy, and dexamethasone can reduce COVID-19 mortality in critically sick patients by about a third. Other evidence from SARS and MERS suggests, however, that corticosteroids may aggravate lung damage due to virus clearance delays [227]. The aforementioned inconsistencies are linked to the peculiarities of critical-condition dynamics, hyperergic and hypoergic phases of systemic inflammation [5], as well as the possibility of SARS-CoV-2-induced damage to the adrenal glands, the formation of vascular thrombi, and the consequences of microcirculatory disorders. In any case, the practicality of employing hormonal therapies in COVID-19 should be assessed by examining the pathogenetic picture of the disease, as well as the functioning state of the HPA, which necessitates a more extensive clinical investigation from an evidence-based medicine approach.

## 3. COVID-19 and Typical Pathological Processes

### 3.1. General Characteristics of Canonical Inflammation

The universal (general pathological) response of the body to damage is inflammation. Canonical (classical) inflammation is the body’s response to pronounced local damage [228,229]. The main canonical inflammation is the presence of a focus of inflammation. Classic inflammation is aimed at localizing, stopping the damaging factor, and subsequent restoration of the damaged tissue. In case of severe damage, the processes of the focus of inflammation can be supplemented by pro-inflammatory reactions in distant organs (primarily, the liver, lymphoid organs, and in the organs of the neuroendocrine system). They develop in response to secondary activation influences and the action of damage factors with a lower intensity than in the inflammation focus. SIR content is determined by these processes, which are primarily intended at giving resources for the inflammatory focus.

**Figure 2:**
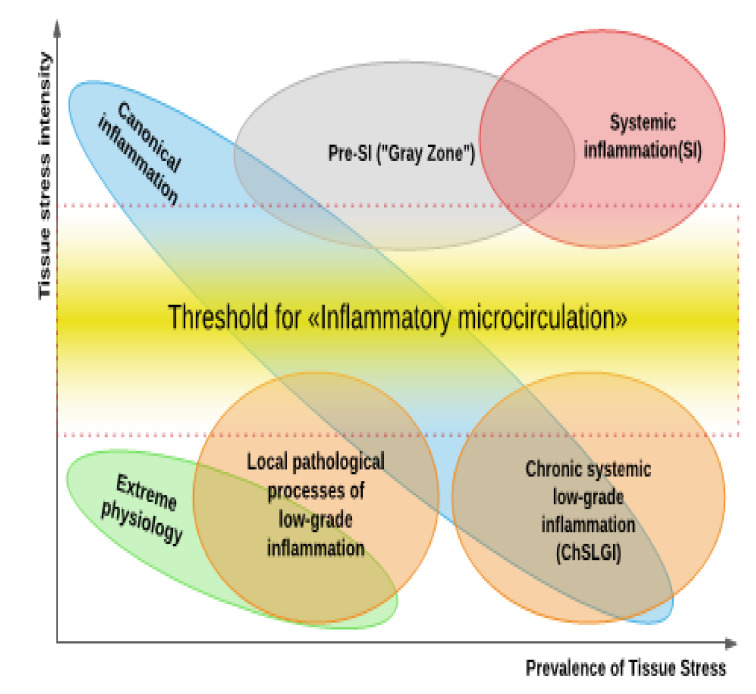
Tissue stress and general pathological processes. Three “large” general pathological processes can be distinguished based on the ratio of intensity (the ordinate in the Figure) to prevalence (the abscissa) of damaging factors initiating a “response” in the form of tissue pro-inflammatory stress—a common pathogenetic basis of all pathological processes (classical inflammation, SI, and ChSLGI). The “inflammatory microcirculation”—a reaction of microvessels that causes a clinically significant exudative reaction and enormous migration of leukocytes into a damaged area with the establishment of the inflammation focus—is the key differentiating phenomenon for their separation. (1) The existence of an inflammation focus (upper-left corner in Figure 2), as well as, in some cases, the systemic manifestations of tissue stress at a subthreshold level for “inflammatory microcirculation”, characterizes classic inflammation. In most cases, these systemic reactions are protective and targeted at providing resource support for the inflammation’s focal point (immune response, the acute-phase response of the liver, forced leuko-cytopoiesis, neuroendocrine stress, and mobilization of metabolic reserves). (2) Systemic inflammation (SI) is characterized by shockogenic systemic “inflammatory microcirculation” and the presence of a transitional “gray” zone, in which it is impossible to prove or refute the presence of SI in situations of the “pushing” variant development (upper-right corner of the Figure). (3) Parainflammation (low-intensity inflammation): can be local (see the figure in the lower-left corner of Figure 1) or can spread throughout the body (see ChSLGI) (lower-right corner of Figure 1). The signs of endotheliosis and, in certain circumstances, latent microcirculatory disorders can be disclosed within these types of tissue pro-inflammatory stress.

However, not all pro-inflammatory processes in humans fit into the canvas of the Procrustean bed of classical ideas about inflammation. Reactions to low-intensity traumas or, conversely, high-intensity systemic injuries are examples of these processes (Figure 2). In theory, they do not need to concentrate on typical inflammation. Due to the lack of a comprehensive response to this dilemma, the idea of these processes marginal in relation to classical inflammation began to be destined in the form of conceptual syndromes. Para-inflammation processes, on the other hand, can describe not only pathological but also many normal and severe physiological processes, as will be illustrated below. As a result, the question arises of whether it is necessary to develop a theory of general pathological processes that is more adaptable to the current realities of practical medicine.

### 3.2. Cellular Pro-Inflammatory Stress as an Elementary, Integral Functional System of Pathological Processes

Cellular pro-inflammatory stress can be defined as follows: “Typical morphofunctional changes in cells in response to damage or threat of damage, aimed at adaptation of the cell, tissue/organ, and organism to the action of damaging factors of various nature”. Cellular stress is a universal biological phenomenon that occurs across all species and levels of development. Cellular stress is composed of several various processes that are all linked together to form a whole, such as [5]:

1. Oxidative stress.
2. Cell response to the DNA damage.
3. Mitochondrial stress, including mitochondrial unfolded protein response (UPRmt).
4. Stress of the endoplasmic reticulum (ER), including calcium-dependent mechanisms and UPRER.
5. Response of inducible heat shock proteins (HSPs), including their participation in the UPR.
6. Inhibition (during cell growth) or intensification of autophagy processes (utilization of altered organelles and macromolecules) and other manifestations of lysosomal stress.
7. Education by inflammasomes.
8. Formation of stress, non-coding microRNAs (miRNAs).
9. Formation of an intracellular network of signaling pathways of cellular stress.
10. Formation of pro-inflammatory receptor and secretory cell phenotype.

Some universal processes and principles of cellular stress organization have profound evolutionary roots and can already be discovered in prokaryotes [230,231,232,233]. At the same time, the “general stress response,” defined as the cell’s ability to defend itself not just from a single stress trigger but also from a variety of seemingly unrelated causes [229,230], is possibly the most fundamental biological trait of this system.

The main effects of pro-inflammatory cellular stress are (1) complete restoration of the cell’s structure and function, as well as the transition of the cell to a new qualitative physiological state, such as differentiation from proliferation; (2) chronicity of stress and the formation of stable morphofunctional changes, such as cellular aging, hypotrophy, hyperplasia, and metaplasia; (3) cell death as a result of apoptosis or programmed necrosis; (4) cell malignancy and tumor growth [4].

Complex epigenetic programming and the intertwining of many inducible signaling pathways, the protein parts of which are constantly undergoing multiple post-translational changes, generate proinflammatory cell stress [234]. At the same time, various extracellular and intracellular stress signals can be activated in different cells (i.e., common small G-proteins Ras, antiapoptotic proteins of the Bcl-2 family, AuTophaGy proteins, heat shock proteins, chaperones, collector type protein kinases, and universal transcriptional cellular stress factors as NF-KappaB, p53, AP-1, HIF, HSF, c-Fos, Egr-1, XBP1) [5]. Every day, roughly 10^4^–10^5^ genome damages of various types occur in each normally functioning human cell, the majority of which is repaired by the DNA-damage response [235]. The same signaling molecules can be activated in a variety of ways and engage in a variety of processes, either synergistically or competitively. The interaction of the p53 and NF-KappaB pathways can thus be controlled in a reciprocal manner [236,237]. For example, NF-KappaB is not activated at relatively low levels of oxidative stress, but p53-mediated DNA repair, cell cycle arrest, or apoptosis of irreversibly damaged proliferating cells can be observed. A further increase in the level of oxidative stress activates NF-KappaB and suppresses p53-induced cell apoptosis, resulting in higher cell resistance to oxidative stress and pro-inflammatory activity [237,238]. The transcription factor Nrf2 (signaling pathway Keap1/Nrf2/ARE) is also activated in response to oxidative stress, which initiates the synthesis of a wide spectrum of antioxidant and anti-inflammatory compounds [239]. Cellular stress is thus controlled and reversible thanks to these processes. Insulin and numerous growth hormones activate the first-class PI3K/Akt (protein kinase B)/mTOR signaling pathway [240]. It is also turned on in cancer cells [241]. AMPK (AMP-activated protein kinase) is activated in response to ATP deficit or increased oxidative stress, which leads to the activation of lipolysis, proteolysis, and the autophagy processes, as well as the activation of NF-KappaB [242,243]. Under certain conditions, mTOR can operate as a double-edged sword, assisting in both the replication and release of virions from cells [244].

As a result, cellular stress is a complicated and paradoxical phenomenon. It encompasses several stages of development and resolution that might present themselves in a mosaic form in distinct cell populations/subpopulations under situations of networked intercellular interactions of tissue stress.

### 3.3. Tissue Stress as a Common Basis of Pathology and Extreme Human Physiology; Non-Canonical Variants of Inflammation

The presence of alteration is not always a sign of pathology. It is well recognized that various markers of tissue damage can be seen in small concentrations in the blood of healthy people. Furthermore, in clinically healthy people, small increases in CRP might be seen, which are linked to FFA levels in blood plasma [245]. The presence of inflammasomes and autophagosomes in the intestinal epithelium is required to keep the intestinal mucosa in a physiological state [246,247]. Skeletal muscle can produce and express many cytokines that have autocrine, paracrine, or endocrine effects [248]. Moreover, IL-6 can increase up to 100 times in the bloodstream during exercise [249]. Many signaling pathways of pro-inflammatory stress are involved in both normal and pathological tissue growth [250,251,252]. Thus, pro-inflammatory stress cannot be associated with pathology alone. However, under physiological conditions, these processes are usually time- and spatial-limited, and their severity is regulated by a range of regulatory feedback systems. Simultaneously, the symptoms of moderate SIR can be used to identify transitional states (the “gray zone”) between norm and pathology. Thus, except for the influence of acute and chronic disorders, those who have experienced mental trauma as a kid have greater blood concentrations of TNF-, IL-6, and CRP in adulthood than people who have not had similar injuries [253].

The cumulative accumulation of low-intensity systemic damage factors with aging and metabolic dysfunction generates a qualitative leap in parainflammation processes, leading to the formation of allostasis and the development of ChSLGI, which underpins many chronic pathologies comorbid for COVID-19. Tissue macrophages are the predominant pro-inflammatory cells in ChSLGI, and as the process progresses, they shift towards the functional pole M1 (“classic type of macrophage activation”) [254,255]. Endothelial dysfunction and latent microcirculatory abnormalities are, in turn, the main causes of ChSLGI. Atherosclerosis begins with endothelial dysfunction [256]. As a result, it increases the risk of atherothrombotic injury and contributes to the development of cardiovascular disease [257]. At the same time, in terms of various signs that differ from inflammation of the classic type, atherosclerosis is a more common form of a typical pathological process [5]. Pathological changes in microcirculation play a critical role in the development of chronic organ dysfunction, which not only predisposes to further organ damage but also raises the risk of future macrovascular events [258]. A decrease in the density of perfused microvessels, death of endothelial cells, hypertension, numerous metabolic, endocrine, and immune disorders, tissue hypoxia, and dysregulated angiogenesis all contribute to the multifactorial etiology of ChSLGI [259].

Local low-grade inflammation processes can progress to the development of classical forms of inflammation focus, mainly of mixed productive and exudative–fibrinous type. Diabetic nephropathy [260,261,262] and non-alcoholic fatty liver disease [263,264,265,266] are two conditions that show these alterations.

In turn, SI occurs as the result of a relatively gradual transformation of classical inflammation (the “pushing” variant) into another pathological system, where the system-forming factor is no longer latent but critical for microcirculatory life disorders, or after a sudden crushing effect of high power systemic alteration factors (the “breakthrough” variant) [5]. Thus, we define this state as SI, characterizing it as “typical multisyndrome, phase-specific pathological process, evolving at systemic injury and characterized by the total inflammatory reactivity of the endotheliocytes, plasma and blood cell factors, connective tissue, and, at the terminal stage microcirculatory disorders in vital organs and tissues”. At the same time, in the more common version of “pushing”, the process goes through the stage of pre-systemic inflammation, which is reversible and relatively sensitive to intensive therapy [4]. The development of this process in COVID-19, as a rule, corresponds to this variant of the SI dynamics.

### 3.4. Possible Connection of COVID-19 with the Mechanisms of Cellular Stress and General Pathological Processes

COVID-19 induces not only local oxidative stress (in the area of inflammation) but also makes it systemic. At the same time, reactive oxygen species can cause damage to numerous tissues, either directly or indirectly [267]. Reducing oxidative stress may have a beneficial effect in the early stages of viral infection by preventing the viral protein from binding to host cells through ACE2 [268]. At later stages of the disease, systemic oxidative stress is interrelated with the formation of inflammasomes and with the formation of an inflammatory phenotype of the vascular endothelium and intravascular leukocytes [269,270]. However, the therapeutic potential of antioxidants in COVID-19 [270] may be inferred from lessons learned from prior trials using antioxidants for sepsis. The emergence of oxidative stress and mitochondrial failure in COVID-19 could be linked to patient aging [271].

The exact mechanism by which SARS-CoV-2 targets and manipulates the p53 pathway is still unknown. SARS-CoV-1, on the other hand, has more detailed information. This suggests that SARS-CoV-2 is involved in suppressing p53-dependent antiviral responses [272]. By creating huge amounts of unfolded or misfolded proteins, many viruses generate ER stress in host cells [273]. Viruses can directly control UPR responses with their proteins, which can decrease the host’s antiviral response and is a key aspect of the coronavirus–host relationship [274]. Indeed, data suggest that ER stress and UPR activation are important aspects of CoV virus–host cell interactions [275]. The virus finds the mechanisms it needs to reproduce inside the cell, such as a protein quality control system that includes HSPs, which, in addition to their physiological roles, assist in viral protein stabilization. Many Hsp90 inhibitors have recently been employed in cancer treatment. Several pieces of data suggest that these inhibitors could also be used as antiviral therapies [276]. Many host stress miRNAs may be modulated by pathogenic human coronaviruses, including SARS-CoV-2, to enhance viral replication and/or prevent immune responses [277]. These findings show that miR-26a-5p, miR-29b-3p, and miR-34a-5p play a non-random role in endothelial dysfunction and inflammatory response in individuals with SARS-CoV-2 infection [278]. Even with these incomplete data, SARS-CoV-2 regulation of numerous mechanisms of cellular stress systems appears to be a possibility.

In eukaryotes, autophagy is a fundamental cellular mechanism that degrades damaged cytosolic proteins, intracellular pathogens, and malfunctioning organelles via the vacuole or lysosome [279]. It serves as the initial line of defense against invading pathogens such as viruses. Autophagy is thought to be the fundamental mechanism of SARS-CoV-2 utilization inside cells [280]. NSP6, a common CoV component, increases autophagosome production but limits their expansion and prevents them from maturing into autolysosomes, potentially favoring viral replication and evasion of cellular protection [281,282]. The NLRP3 inflammasome [283] is a molecular platform that promotes inflammation by cleaving and activating critical inflammatory molecules. In monocytes, SARS-CoV-2 infection activates the NLRP3 inflammasome [283,284], suggesting that NLRP3 could be a marker of disease severity and a possible therapeutic target for COVID-19 [283,285].

Taken together, these results suggest that SARS-CoV-2 can affect all key components of pro-inflammatory cellular stress.

**Figure 3:**
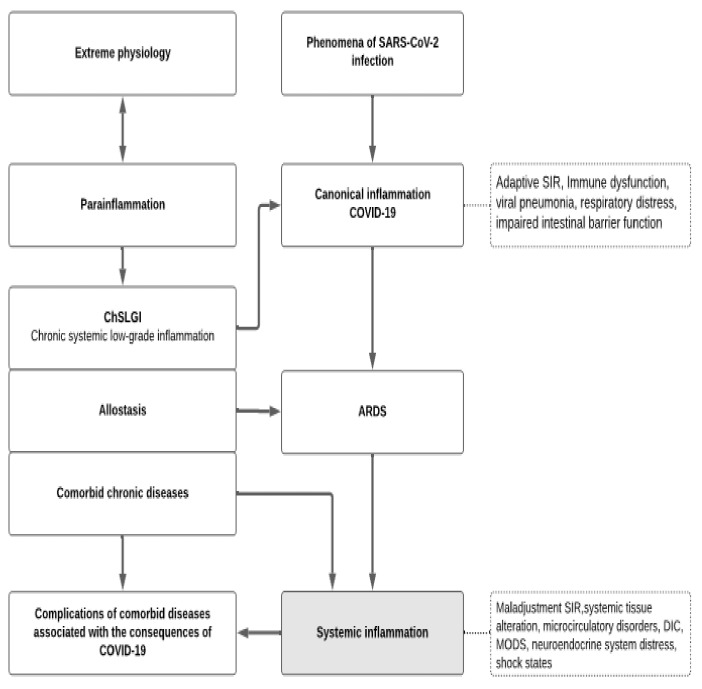
The relationship of general pathological processes in COVID-19. Two interrelated processes are depicted in the heuristic diagram of COVID-19 pathogenesis. In the left part of the scheme, the development of comorbid chronic pathologies, risk factors for SARS-CoV-2 infection, and chronic systemic low-grade inflammation (ChSLGI), which is a common, typical pathogenetic basis of chronic pathologies (in the form of allostasis), as well as comorbid chronic diseases are depicted. ChSLGI is linked to pro-inflammatory tissue stress, a subthreshold for the formation of focus of classical inflammation processes, and life-threatening “systemic inflammatory microcirculation”. In the center, the transformation of canonical pneumonia into Acute Respiratory Distress Syndrome (ARDS), which is already a sign of systemic inflammation (SI), is described. The source for ChSLGI development and stabilization (in the form of allostasis) are the extreme physiological processes in which tissue stress has limitations in its severity, time, and prevalence in the body.

Overall, summarizing Section 3, it is proposed that the pathogenesis of COVID-19 is based on four general pathological processes (Figure 3):

(1) ChSLGI as the basis for comorbid pathologies that are risk factors for critical complications. These pathologies include morbid obesity, metabolic syndrome, and type 2 diabetes, which are known risk factors for the development of critical conditions in ChSLGI [18,22,177,222] and should be considered in Cov infections. The accumulation of aberrant metabolites and their transport forms (including modified lipoproteins) in the blood, latent microcirculation disorders, an increase in the pro-inflammatory phenotype of many cell types, and the dysfunction of neuroendocrine and paracrine regulation of organs and systems are all driving factors in these pathologies.
(2) Inflammation of the classical type, mainly on the territory of the respiratory and intestinal organs, which forms a functional barrier that limits the spread of the virus in the body. The insufficient efficiency of canonical inflammation determines the possibility of generalization of viruses in the body (an additional factor of systemic alteration), excessive production of cytokines (as factors of excitotoxicity), and the development of noncardiogenic pulmonary edema (ARDS).
(3) Systemic inflammation, which is a typical critical complication of COVID-19, the clinical manifestations of SI are complexes of resuscitation syndromes. For the SI development, the integral factors of systemic alteration must reach a level sufficient for the development of the “systemic inflammatory microcirculation” phenomenon.
(4) The possible influence of transferred SI on the course of chronic comorbid diseases requires additional prospective studies.

These processes are interconnected through the universal mechanisms of cellular and tissue pro-inflammatory stress. They share common factors of alteration, yet they work in diverse circumstances. At the same time, ChSLGI processes are factors that provoke dysfunction of canonical inflammation and immune response, predetermining the development of SI. In turn, the consequences of systemic damage in COVID-19 predetermines the emergence of long-term complications associated with the instability of ChSLGI allostasis in surviving patients.

## 4. Conclusions and Future Perspectives

Problems such as the COVID-19 pandemic test not only the state of practical health care in various countries [2,286] but also the state of fundamental medicine. The fundamental laws of pathology reflect the models of general pathological processes (pathological systems). We feel that the idea of typical pathogenic processes is now outmoded, particularly in terms of the characteristics of inflammatory processes that go beyond conventional concepts of inflammation. The models of conceptual syndromes, such as SIRS, CRS/CSS, thrombo-inflammatory syndrome [287,288], and others, are required to fill these gaps. In addition to establishing specific standard protocols for patient management (not all conceptual syndromes have such), formal constructions of conceptual syndromes serve as theoretical generalizations. However, they can only show particular aspects of a complex phenomenon, not a whole picture of a pathological process, especially when numerous processes with distinct dynamics and nature interact. The latter, in particular, dictates how comorbidity affects COVID-19’s clinical trajectory.

Meanwhile, the wealth of data gathered in clinical and experimental studies already dictates the need to modify existing theoretical generalizations in the fields of pathological physiology, integrative immunology, and general pathology. This is true not only for individual general pathological processes but also for the need to develop a general theory of pathological systems that considers the unity of universal mechanisms of cellular and tissue pro-inflammatory stress, which is present not only in diseases of various types but also in many physiological processes. Otherwise, when attempting to answer specific clinical difficulties, one will be forced to “stumble at every step” over unsolved problems of a higher order, frequently misrepresenting the particular as the general and the consequence as the cause. This is true in large part for the pathogenetic therapy of complex diseases such as COVID-19. In this context, the appearance of this impact, rather than the very limited favorable effect of anti-cytokine therapy for COVID-19, may be surprising. With the current validity of anti-cytokine therapy for COVID-19, apparently, the positive effect can be explained by the phenomenon of “Wisdom of nature”, namely, the ability of the body’s regulatory systems to suppress potentially harmful external influences and enhance beneficial ones. Pathogenetic therapy of critical conditions, including in COVID-19, should probably be multi-targeted, affecting various links of the pathological process.

True multi-target activity is thought to be the foundation for pharmacological polypharmacology, which is induced by simultaneous in vivo interactions with numerous targets [289,290,291]. Due to the presence of many channels of viral transmission, various mechanisms of virus impact on immune regulation and antiviral protection, and the multifactorial nature of COVID-19 disease pathogenesis, polypharmacology may be effective in the treatment of COVID-19 [292].

Thus, small molecules with multitarget activity are of increasing interest for interfering with derailed cellular signaling pathways or perturbed target networks implicated in complex multifactorial diseases such as COVID-19 [293,294,295,296]. In this situation, multi-target therapy could be used to provide a synergistic antiviral, immunomodulatory, and anti-inflammatory impact at the same time. Around 4000 clinical trials on COVID-19 have been conducted worldwide [297], employing small molecules as single or combination treatments with various antiviral medicines.

To date, several studies have begun to look at the active compounds found in medicinal plants used in Traditional medicine since many of them have a long history of antiviral activity [290,296,298,299,300,301,302]. Numerous groups have used in silico computational approaches, drug-like filters, and toxicity studies to screen small drug-like molecules from a dataset of phytochemicals with antiviral activities [299], and the number of multi-target natural compounds were predicted to suppress the activity of three or more major drug targets simultaneously [296,301,303,304]. Thus, compounds that targeted five, four, and three drug targets and exerted anti-COVID-19 activities by multi-component and multi-target action were found in the studies of B Naik et al. (2020) and L-Q Gao (2020) [296,303]. Due to their capacity to control immune and inflammation-related targets and pathways, phytochemicals from Toujie Quwen Granules (TQG) and Huashi Baidu formula (HSBDF) were later recommended as promising drugs [305,306]. Likewise, among 7000 Ayurvedic anti-tussive medicines, anti-viral phytochemicals, and synthetic anti-virals, several molecules that were strongly bound to SARS-CoV-2 MPro (i.e., δ-viniferin, myricitrin, taiwanhomoflavone A, afzelin, biorobin, and hesperidin) [304] have been revealed. Diacerein (diacetylrhein), an anthraquinone derivative with multi-target action that controls hyperinflammatory conditions through cytokine suppression and anti-platelet aggregation activity, and maybe effects on viral infection and replication, has also been proposed for therapeutic application [307]. Based on these, it could be suggested that active phytochemicals from medicinal plants could equip the management strategy against COVID-19-a global contagion [300,308].

Furthermore, the computational docking results revealed that several known and newly synthesized chemical compounds had high inhibitory binding affinities with the docked SARS-CoV-2 proteins, suggesting that they could be useful lead compounds for the design and synthesis of new anti-COVID-19 agents [304,309,310]. A combination of in silico analysis of immune system protein interactome networks, single-cell RNA sequencing of human tissues, and artificial neural networks was undertaken in one of the recent (2021) studies to reveal potential therapeutic targets for drug repurposing against COVID-19 [297]. As a consequence, 47 approved drugs, 25 under-investigation compounds, and 50 experimental compounds with a strong affinity for 15 possible therapeutic targets were discovered and recommended for further research [297].

In the fight against the pandemic, protein target-oriented drug management is not less important, and various molecules have been presented as potential therapeutic options for SARS-CoV-2 (reviewed in [311]). Finally, several mesenchymal stem-cell-derived extracellular vesicles carrying miRNA due to their intrinsic properties to modulate the exacerbated cytokines, cell death, and coagulation disturbances present in severe COVID-19 were proposed as a potential multitarget therapy [312]. 

Overall, the current findings underscore the relevance of multi-target therapeutic approaches that take into account the typical patterns of critical condition development, which are promising not just for COVID-19 treatment but also for other CoV infections.

## Figures and Tables

**Table 1:** Differences in the levels of cytokines and acute-phase proteins (ferritin and CRP) in patients receiving intensive therapy (higher concentrations) and with a milder course of COVID-19 (less high).

Significant Differences (*p* < 0.05)	Unreliable (*p* > 0.05) or Insignificant *	Sources
IL2, IL7, IL10, IP10 (CXCL10), MCP-1 (CCL2), MIP-1α (CCL3), TNF-α	IL4, IL5, IL13, Eotaxin, RANTES, IL9, IL12p70, IL15, IL17A, IL1β, IFN-γ	[36]
sIL-2R (sCD25) and IL-6	TNF-α, IL-1β, IL-8, IL-10, CRP	[34,42]
IL-6 and IL-10	TNF-α, IFN-γ, IL-2, IL-4 [36]	[37,49]
IL-6 and IFN-γ (in the later), IL-10, IL-1RA	RANTES (CCL5), GM-CSF, TNF-α	[33]
IP10 (CXCL10) and MCP-1(CCL2)		[39]
TNF-α, IL-10 and IL-6, IP-10, MCP-3 (CCL7), IL-1ra, IL-17A		[40]
CRP, IL-6, Ferritin	IL1β [40], low differences Me *: TNF-α, IL-8, IL-10, sIL-2R [40]; PCT [43]	[41,43,44]
sIL-2R, TNF-α and IL-10, IL-6		[28,35,45,53]
IL-6, IL-10, IL-2, IFN-γ		[46]
IL-6, IL-10	IL-2, IL-4, IFN-γ, TNF-α	[42,47,50,51,52]
IL-6, sIL-2R, IL-8, IL-10, and TNF-α,		[48]
IP-10, MCP-3, IL-1ra		[54]
PCT, TNF-α, VEGF-A, Angpt-2	CRP, IL-6, Ferritin	[55]

Me is the median; IL—interleukin-2; IL10—interferon gamma-induced protein 10; MCP—monocyte chemoattractant protein; MIP—macrophage inflammatory protein; IFN—interferon; TNF—tumor necrosis factor; IL-1ra—interleukin-1 receptor antagonist; RANTES—regulated on activation, normal T cell expressed and secreted; sIL-2R (sCD25)—soluble interleukin-2 receptor; GM-CSF—granulocyte-macrophage colony-stimulating factor; CRP—C-reactive protein; PCT, procalcitonin; VEGF—vascular endothelial growth factor; Angpt—angiopoietin; CXCL—C-X-C motif chemokine ligand; CCL—chemokine (C-C motif) ligand.
